# Interferon-gamma is quintessential for NOS2 and COX2 expression in ER^-^ breast tumors that lead to poor outcome

**DOI:** 10.1038/s41419-023-05834-9

**Published:** 2023-05-11

**Authors:** Robert Y. S. Cheng, Lisa A. Ridnour, Adelaide L. Wink, Ana L. Gonzalez, Elise L. Femino, Helene Rittscher, Veena Somasundaram, William F. Heinz, Leandro Coutinho, M. Cristina Rangel, Elijah F. Edmondson, Donna Butcher, Robert J. Kinders, Xiaoxian Li, Stephen T. C. Wong, Daniel W. McVicar, Stephen K. Anderson, Milind Pore, Stephen M. Hewitt, Timothy R. Billiar, Sharon A. Glynn, Jenny C. Chang, Stephen J. Lockett, Stefan Ambs, David A. Wink

**Affiliations:** 1 Cancer Innovation Laboratory, Center for Cancer Research, National Cancer Institute, National Institutes of Health, Frederick, MD USA; 2 Optical Microscopy and Analysis Laboratory, Frederick National Laboratory for Cancer Research, Leidos Biomedical Research Inc. for the National Cancer Institute, Frederick, MD USA; 3 Center for Translational Research in Oncology, ICESP/HC, Faculdade de Medicina da Universidade de São Paulo; and Comprehensive Center for Precision Oncology, Universidade de São Paulo, São Paulo, SP Brazil; 4 Molecular Histopathology Laboratories, Leidos Biomedical Research Inc. for NCI, Frederick, MD USA; 5 Office of the Director, Division of Cancer Treatment and Diagnosis, NCI, Frederick, MD USA; 6 Department of Pathology and Laboratory Medicine, Emory University, Atlanta, GA USA; 7 Systems Medicine and Bioengineering Department, Houston Methodist Neal Cancer Center, Houston Methodist Hospital and Weill Cornell Medicine, Houston, TX USA; 8 Imaging Mass Cytometry Laboratory, Cancer Research Technology Program, Frederick National Laboratory for Cancer Research, Frederick, MD USA; 9 Laboratory of Pathology CCR, NCI, NIH, Bethesda, MD USA; 10 Department of Surgery, University of Pittsburgh Medical Center, Pittsburgh, PA 15213 USA; 11 Discipline of Pathology, Lambe Institute for Translational Research, School of Medicine, University of Galway, Galway, Ireland; 12 Mary and Ron Neal Cancer Center, Houston Methodist Hospital and Weill Cornell Medicine, Houston, TX USA; 13 Laboratory of Human Carcinogenesis, CCR, NCI, NIH, Bethesda, MD USA

**Keywords:** Translational research, Prognostic markers

## Abstract

A strong correlation between NOS2 and COX2 tumor expression and poor clinical outcomes in ER breast cancer has been established. However, the mechanisms of tumor induction of these enzymes are unclear. Analysis of The Cancer Genome Atlas (TCGA) revealed correlations between NOS2 and COX2 expression and Th1 cytokines. Herein, single-cell RNAseq analysis of TNBC cells shows potent NOS2 and COX2 induction by IFNγ combined with IL1β or TNFα. Given that IFNγ is secreted by cytolytic lymphocytes, which improve clinical outcomes, this role of IFNγ presents a dichotomy. To explore this conundrum, tumor NOS2, COX2, and CD8^+^ T cells were spatially analyzed in aggressive ER–, TNBC, and HER2 + breast tumors. High expression and clustering of NOS2-expressing tumor cells occurred at the tumor/stroma interface in the presence of stroma-restricted CD8^+^ T cells. High expression and clustering of COX2-expressing tumor cells extended into immune desert regions in the tumor core where CD8^+^ T cell penetration was limited or absent. Moreover, high NOS2-expressing tumor cells were proximal to areas with increased satellitosis, suggestive of cell clusters with a higher metastatic potential. Further in vitro experiments revealed that IFNγ + IL1β/TNFα increased the elongation and migration of treated tumor cells. This spatial analysis of the tumor microenvironment provides important insight into distinct neighborhoods where stroma-restricted CD8^+^ T cells exist proximal to NOS2-expressing tumor niches that could have increased metastatic potential.

## Introduction

Estrogen receptor alpha-negative (ER−) and triple-negative breast cancer (TNBC) account for a smaller proportion of breast cancer types but are among the most aggressive malignancies with limited treatment strategies when compared to less aggressive ER + tumors [1]. During the past decade, a significant proportion of cancers have demonstrated elevated NOS2 expression [2], where cancers ranging from melanoma to glioma overexpress NOS2 [3–6]. In breast cancer, increased NOS2 has been reported in >70% of patients [7]. Interestingly, elevated tumor NOS2 expression correlated with P53 mutation and was predictive of poor survival in ER− (Hazard Ratio = 6) but not ER + breast cancer patients [7, 8]. While elevated tumor COX2 expression was also predictive of a poor outcome as defined by HR of 2.45 in ER- patients from the same cohort [9], elevated NOS2/COX2 coexpression was strongly predictive of poor outcome (HR 21) [10]. These results suggest that elevated tumor NOS2/COX2 coexpression drives the progression of aggressive breast cancer phenotypes [10, 11]; however, mechanisms of NOS2/COX2 induction within tumors remain unclear.

Examination of The Cancer Genome Atlas (TCGA) has revealed correlations between tumor NOS2/COX2 expression and interferon-gamma (IFNγ), interleukin-17 (IL17), IL1, and toll-like receptor-4 (TLR4), which are frequently associated with anticancer effects [12]. Interestingly, these associations are contradictory as elevated tumor NOS2/COX2 expression predicts poor clinical outcomes [7, 9, 10]. Recently, elevated NOS2 and COX2 expression was discovered in distinct immune and tumor cells upon treatment with IFNγ and cytokines or TLR4 agonists, consistent with feedforward NOS2/COX2 signaling as previously reported [10, 13]. These results suggest an orthogonal relationship between tumor NOS2/COX2 expression, which could promote distinct tumor microenvironments that contribute to poor clinical outcomes [13].

To explore these possibilities, herein, we show that Th1 cytokines effectively stimulate NOS2 and COX2 expression in tumor cells in vitro. Single-cell RNAseq (scRNAseq) analysis revealed that IFNγ combined with interleukin 1β (IL1β), or tumor necrosis factor-alpha (TNFα) induced higher NOS2/COX2 expression than the cytokines as single agents. In addition, cytokines that were upregulated in high NOS2-expressing ER-breast tumors [7, 10], including IL6 and IL8, were induced by these treatments and correlated with NOS2/COX2 expression. Also, this study reveals a unique synergy between IL1α/β that enhances the expression of NOS2 and COX2. Multiplex spatial imaging revealed clusters of high NOS2 expressing cells proximal to areas of stroma-restricted CD8^+^ T cells that are known to produce IFNγ and suggest a small inflammatory niche at the tumor/stroma interface in these tumors. While COX2 was present in these regions, it was more highly expressed further into the tumor, in immune desert areas with low CD8^+^ T cell penetration. Comparison of distinct sites within the same tumor, or geographic regions between tumors, suggests a spatial and temporal progression of inflammatory sites in areas of restricted lymphoid cells, which progresses to an immune desert in areas of high COX2 expression. These novel observations provide distinct spatial fingerprints of aggressive tumor phenotypes that correlate with decreased disease-specific breast cancer survival [7, 10].

## Results

### TCGA and bioinformatics for tumor NOS2/COX2 expression

**Fig. 1:**
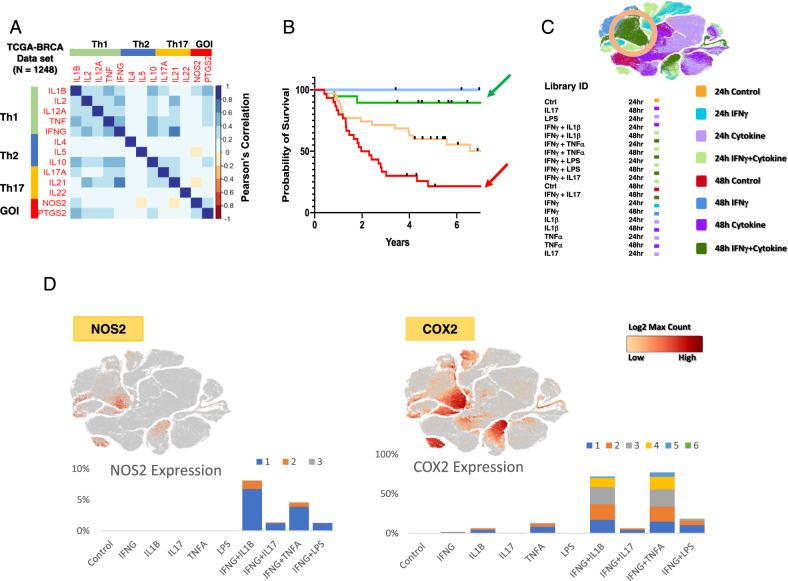
IFNγ and cytokines induce maximal expression of NOS2 and COX2 in ER-breast cancer cells. **A** A heatmap display of Pearson’s correlation analysis of TCGA-BRCA (*n* = 1248) database through the UCSC Xena browser analyzing Th1, Th2, Th17, and two GOI (gene of interest) genes. The heatmap was generated in corrplot (0.92) in R (4.2.1). **B** Survival analysis associated with NOS2_lo_/COX2_hi_ (blue arrow), NOS2_lo_/COX2_lo_ (green arrow), NOS2_hi_/COX2_lo_ (yellow arrow), and NOS2_hi_/COX2_hi_ (red arrow) tumor protein expressions. **C** t-SNE plot (Loupe Browser 6.3.0) of single-cell analysis of MB231 cells treated with single or combination of cytokines IFNγ (100 U/ml), IL1β (10 ng/ml), TNFα (10 ng/ml), IL17 (100 ng/ml), and LPS (10 ng/ml) for 24 and 48 h. Light and dark green color clusters represent 24- and 48-h time points associated with IFNγ + IL1β/TNFα treatment, respectively. The orange circle indicates the highest overlapped cell clustering for the IFNγ + IL1β or TNFα after 48 h treatment. **D** t-SNE plots of NOS2 and COX2 clustering cells. Stacked bar charts show the number of NOS2 and COX2 transcripts per cell in 48 h treatment groups. Transcript per cell data (color code: blue, 1; orange, 2; gray, 3; yellow, 4; cyan, 5; green, 6) were extracted using the R packages (data.table 1.14.2, dplyr 1.0.10, and ggplot2 3.3.6).

Our previous work comparing high and low NOS2 and COX2 (also known as PTGS2) tumor expression revealed associations with a variety of inflammatory markers that are typically associated with antitumor activity [10], which prompted us to explore cytokine regulatory effects on NOS2/COX2 expression in tumor cells and tissues. A correlation analysis was performed using the Xena browser and TCGA database to gain a deeper understanding of the conditions associated with NOS2 and COX2 expressions in ER-breast cancer. The correlation analysis of TCGA-BRCA (breast cancer) revealed several inflammatory pathways associated with increased tumor NOS2/COX2 expression (Fig. 1A) that influenced clinical outcomes (Fig. 1B), including the antitumor-associated IFNγ, TNFα, IL2, IL1, and IL17 pathways (Fig. 1A). Moreover, tumor NOS2/COX2 expressions were positively correlated and Th1 cytokines had the strongest positive association with COX2, whereas IL1β was associated with NOS2 (Fig. 1A). These findings suggest a dichotomy within the tumor microenvironment (TME), where cytokines that are generally associated with favorable outcomes also promote elevated tumor NOS2/COX2 expressions (Fig. 1A), which are strong predictors of poor disease-specific survival in ER- breast cancer patients (Fig. 1B) [7, 9, 10]. To confirm the regulatory roles of these cytokine(s) in the induction of NOS2 and/or COX2 expressions, MDA-MB231 (MB231) breast cancer cells were stimulated with IFNγ in the presence and absence of TNFα, IL1β, IL17, and the TLR4 agonist lipopolysaccharide (LPS). Single-cell RNAseq data revealed that 48 h exposure to IFNγ combined with TNFα or IL1β induced the highest expressions of NOS2 and COX2 (Fig. 1C). Consistent with previous reports in murine tumors [12], high NOS2 expression requires IFNγ and TNFα/IL1, which exhibit a striking difference in cytokines produced during induction of murine macrophages and tumor cells [12]. Moreover, scRNAseq of cytokine-stimulated MB231 cells revealed that a 48 h stimulation with IFNγ in the presence of IL1β or TNFα induced the highest NOS2 and COX2 expressions, which clustered in the same regions of the t-SNE plot (Fig. 1C, D). These results confirm high NOS2 and COX2 expression is induced by IFNγ combined with IL1β or TNFα as shown in the TCGA analysis. As predicted, scRNAseq analysis of IFNγ + IL1β/TNFα revealed an increase in the transcript levels of NOS2 and COX2 in treated MB231 cells. The number of NOS2 transcripts ranged from 1 to 3, whereas COX2 transcripts had a significantly wider range (Fig. 1D). While less than 9% of the cells contained NOS2 transcripts, up to 40% of the cells contained COX2 transcripts (Fig. 1D). NOS2 expression required IFNγ, whereas COX2 expression was weakly induced by IL1β or TNFα alone (Fig. 1D). Nonetheless, in a manner similar to NOS2, the strongest COX2 expression occurred by IFNγ + IL1β/TNFα. In addition to cytokine stimulation, NOS2/COX2 feedforward signaling could also promote elevated NOS2/COX2 expression (Fig. 1A), as previously reported [10].

**Fig. 2:**
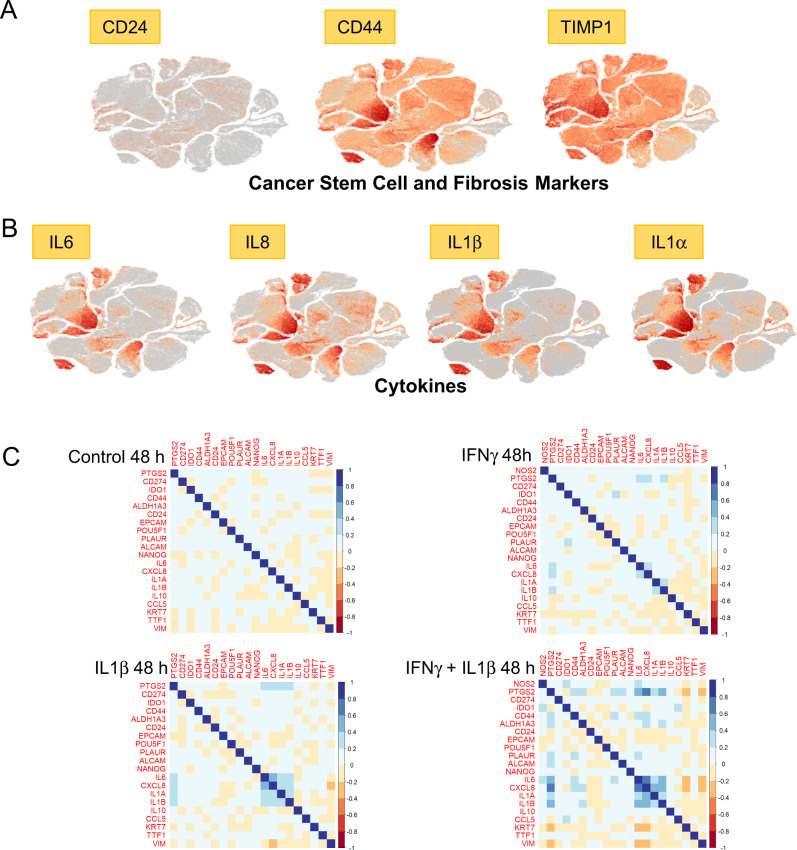
t-SNE plot and correlation analyses. **A** t-SNE plots of cancer stem cell (CD24/CD44) and fibrosis (TIMP1) markers and **B** Th1 cytokine (IL6, IL8, IL1β, IL1α) markers, which clustered in the same regions as NOS2/COX2 shown in panel **A**. **C** Correlation analysis of selected cytokines, cancer stem cell markers, and metastatic-associated genes. Data were extracted from 48 h Control, IFNγ, IL1β, and IFNγ + IL1β treated groups in corrplot (0.92) in R (4.2.1). NOS2 was not expressed in Control and IL1β treated cells and is therefore not represented in the correlation analysis.

Cancer stemness markers have been reported in cell populations with elevated CD44 and reduced CD24 expression levels that exhibited the same drug-resistant histopathological features of the derived tumor when injected in mice at very low concentrations [14]. Herein, t-SNE plot analysis of CD44/CD24 expression revealed distinct clustering patterns (Fig. 2A) defined by increased CD44 and reduced CD24 levels in high NOS2/COX2-expressing clusters after 48 h treatment with IFNγ + IL1β or TNFα. This pattern is suggestive of increased cancer stemness [14] and is consistent with earlier reports of elevated CD44 levels in high NOS2-expressing ER- breast tumors [7, 15]. The expression of tissue inhibitor metalloproteinase-1 (TIMP1), a fibrosis marker [16], was similar to CD44, indicating that their expressions were likely controlled by the same upstream regulator (Fig. 2A). To explore the expression relationships between Th1 cytokines, we analyzed the clustering patterns of IL6, IL8, IL1α, and IL1β (Fig. 2B). The clustering patterns of these cytokines are highly similar and strongly overlap after 48 h stimulation with IFNγ + IL1β/TNFα (Fig. 2B). Moreover, examination of other cytokine-induced genes revealed a strong association with IL1α/β (Fig. 2C) and are consistent with observations demonstrating that circulating IL1β predicts poor survival [17]. Together, these findings support the TCGA-BRCA correlation analysis shown in Fig. 1A.

### Spatial identification of NOS2 and COX2 niches

**Fig. 3:**
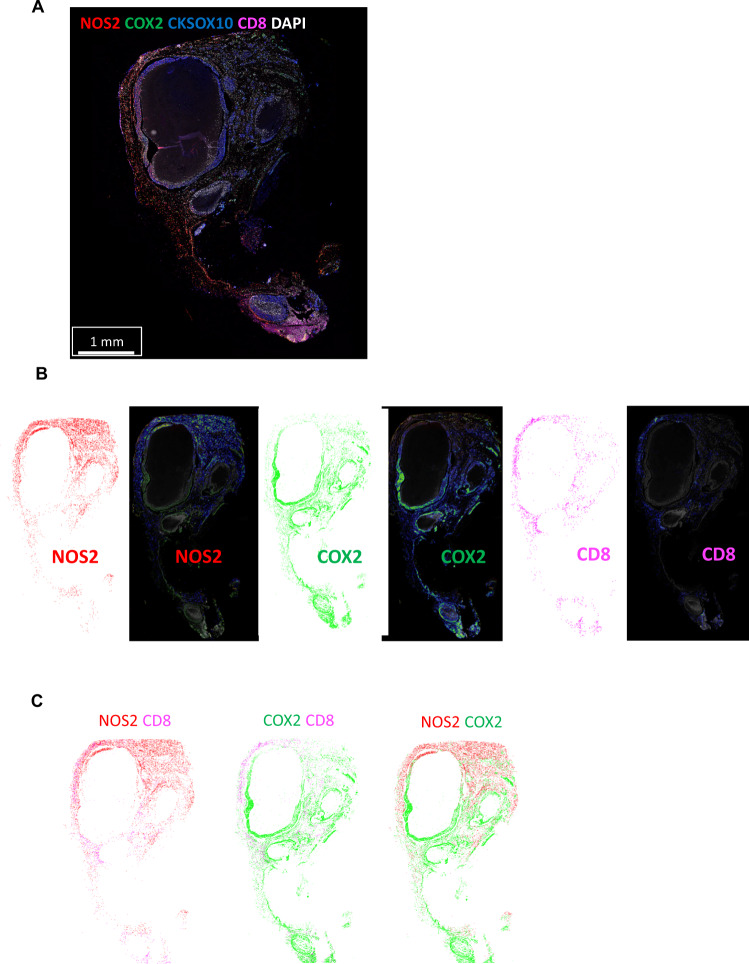
Tumor NOS2/COX2 and CD8^+^ T cells occupy unique areas in the tumor microenvironment. **A** Multiplex fluorescence of an ER-/HER2 + breast tumor showing NOS2 (red), COX2 (green), CKSOX10 (blue), CD8^+^ T cells (magenta), and DAPI (white). **B** Spatial distribution (left) and density heat maps (right) of NOS2, COX2, or CD8^+^ T cells. Spatial distributions reflect a positive detection of the markers within 25 µm diameter areas independent of amount. Density heat maps provide a visual quantitation reflected by color gradation (low-high) blue, green, yellow, orange, and red of the biomarker protein expressions. **C** comparison of NOS2/CD8, COX2/CD8, and NOS2/COX2 spatial distribution combinations.

The above in vitro findings demonstrate that IFNγ is a key regulator in the induction of NOS2/COX2 expression in ER- breast tumor cells, which raises the question of the origin of IFNγ secretion in tumor tissues. Cytotoxic lymphocytes release IFNγ and are associated with improved survival in TNBC and other cancer types [18–20]. In contrast, elevated tumor NOS2/COX2 expression promotes disease progression and is strongly predictive of poor disease-specific breast cancer survival [7, 9, 10]. These findings implicate a dichotomy where antitumor lymphoid-producing IFNγ cells could induce pro-tumor NOS2/COX2-expressing cellular niches, which may be due to heterogeneity within the TME. To explore this hypothesis, the spatial proximity and relationship between CD8^+^ T cells that produce IFNγ, and tumor NOS2/COX2-expressing cells was examined in 21 ER- breast tumors (including TNBC (*n* = 14) and HER2/neu^+^ (*n* = 7) phenotypes) using multiplex spatial imaging. Fluorescence imaging enables the visualization and quantification of cellular neighborhoods at the single-cell level. High NOS2 and COX2-expressing cells were observed in distinct regions of the TME, as depicted in Fig. 3A. Spatial distribution and density heatmap analyses of the whole tumor reveal distinct regions of NOS2, COX2, and CD8^+^ T cells where NOS2- and COX2-expressing cells (Fig. 3B, C) were observed in separate neighborhoods. In addition, higher CD8^+^ T cell densities were observed near NOS2-expressing cells, while lower densities were identified near COX2-expressing clusters (Fig. 3B, C). Thus, spatially distinct NOS2 and COX2-expressing cells in relation to CD8^+^ T cells in aggressive breast tumors suggest an association between CD8^+^ T cells and cytokine-induced NOS2/COX2 niches that influence clinical outcomes.

### NOS2 and COX2 fluorescence intensity

**Fig. 4:**
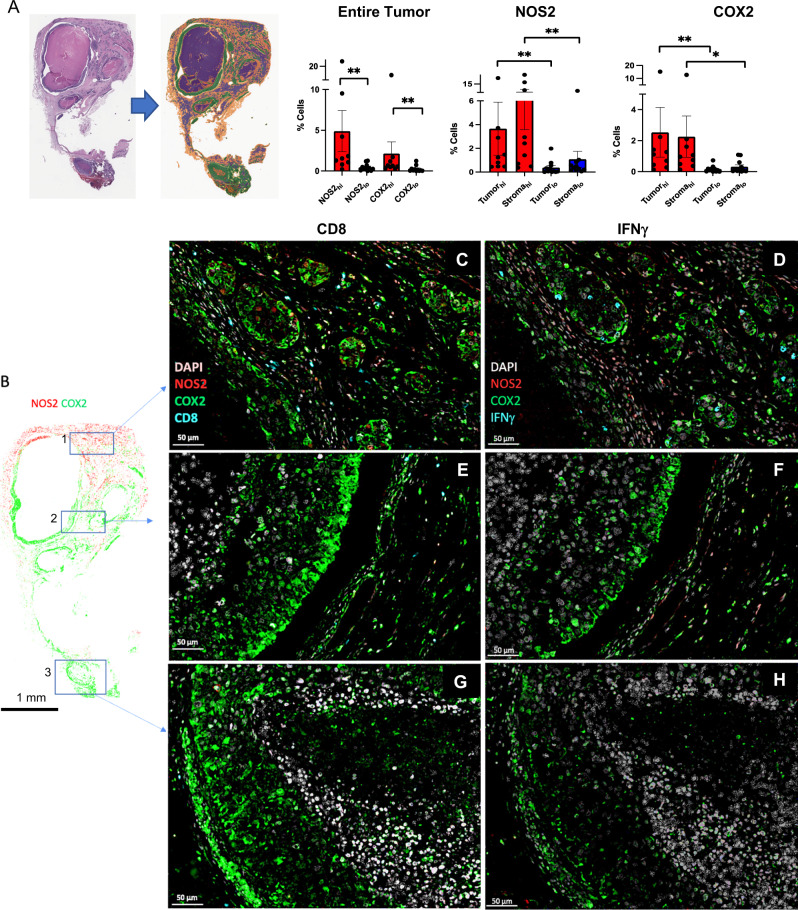
Correlation of pathology scoring and single-cell fluorescence intensities. **A** H&E-stained section (left) fused with a serial fluorescent image (right). H&E sections were evaluated by a pathologist who defined areas of necrosis (purple), viable tumor (green), and stroma (orange). The %NOS2/COX2-expressing cells in the entire tumor as well as tumor and stroma, are shown. **B** Areas in the spatial distribution highlighted by blue boxes labeled 1 (top), 2 (middle), and 3 (bottom) reflect a progression from (1) inflamed regions of stroma-restricted CD8^+^ T cells to (2) cold regions with reduced stroma-restricted CD8^+^ T cells and (3) cold immune desert tumor core regions lacking CD8^+^ T cells. Spatial analyses of NOS2/COX2 expression in these boxed areas as well as CD8^+^ T cells or IFNγ, are shown at 50 μm magnification. Registered images in the inflamed region designated in box 1 shows **C** stroma-restricted CD8^+^ T cells (cyan) or **D** IFNγ expression (cyan) near NOS2-expressing cells (red). Analyses of cold regions designated in box 2 show high COX2 expression and low NOS2 expression with **E** limited CD8^+^ T cells and **F** limited IFNγ. Analyses of immune desert tumor core regions associated with box 3 show high tumor COX2 expression with abated levels of **G** CD8 + T cells and **H** abated IFNγ expression.

The NOS2/COX2 expressions in ER- tumors previously scored as NOS2/COX2 high (hi) or low (lo) by routine immunohistochemistry (IHC) grades of 1–4 [7, 9] were analyzed for NOS2/COX2 fluorescence intensity at the single cell level using multiplexed fluorescence imaging, which provides spatial information at the single-cell level in regions including necrosis, stroma, and viable tumor. Viable tumor and stroma regions were annotated by a Veterinary Pathologist on H&E images (QuPath) [21] and fused with NOS2/COX2 fluorescent expression using HALO software (Fig. 4A). NOS2 and COX2 fluorescent intensities were determined for each tumor using real-time tuning in HALO software, and then mean intensities and standard deviations (SD) were determined. Threshold intensities for weak, moderate, and strong expression levels were determined by adding 2, 4, or 6 SD, respectively, to the mean intensity threshold setting. NOS2/COX2 fluorescent intensities of the entire tumor quantified from these thresholds (Supplemental Fig. 1A) were consistent with the original IHC Pathologist scored NOS2/COX2 expression levels previously reported [7, 9]. When stratifying for tumor vs stroma, NOS2/COX2 tumor expression with strong/moderate signal intensity was significantly elevated in NOS2/COX2 high-expressing tumors (Supplemental Fig. 1B). In contrast, NOS2 weak signal intensities were significantly elevated in the stroma but not tumor, while COX2 weak signal intensity was higher in tumor but not stroma (Supplemental Fig. 1B). NOS2 and COX2 feedforward signaling [10] has been shown to maintain their expressions. A potential linear relationship between tumor NOS2 and COX2 in these tumors was examined using Pearson’s correlation coefficient, which revealed linear correlations between NOS2 and COX2 expression at strong, moderate, and weak intensities (Supplemental Fig. 1C). Together, these results support NOS2/COX2 feedforward signaling as shown in Fig. 1A, and as previously reported [10].

### Spatial correlations of NOS2, COX2, and CD8^+^ T cells

Figure 4A demonstrates significant increases in the %cells with elevated tumor NOS2/COX2 expression in the entire tumor as well as tumor and stroma regions. Further examination of NOS2 and COX2 spatial distributions revealed that NOS2^+^ cells are clustered at tumor margins or in the stroma (Fig. 4B–D). While COX2 expression was observed near NOS2-expressing cells in some regions, COX2^+^ cells were densely clustered in distinct areas deeper into the tumor core as well as in immune desert regions of the tumor (Fig. 4E–H). Tumors scored as NOS2_lo_/COX2_lo_ exhibited sporadic low-density NOS2 and COX2 foci, where few or no higher-intensity foci were observed. In contrast, NOS2_hi_/COX2_hi_ tumors exhibited numerous, spatially distinct, high-expressing NOS2 and COX2 foci, with NOS2 clusters at the tumor-stroma interface (Fig. 4C, D). In contrast, boxes 2–3 show COX2 clusters extending deeper into the tumor core (Fig. 4E–H). Thus, NOS2 and COX2-expressing cells are spatially localized in distinct inflammatory regions of the tumor.

As depicted in Figs. 1D and 2C, IFNγ is necessary for optimal NOS2/COX2 expression in MB231 cytokine-treated cells. Lymphoid cells, including CD8^+^ T cells, are a source of IFNγ secretion [20]. Recent studies have demonstrated a key role in the spatial orientation of CD8^+^ T cells for improved survival in TNBC [22]. Penetration of CD8^+^ T cells into the tumor core defined a fully inflamed tumor that was predictive of improved TNBC patient survival [22]. In contrast, limited CD8^+^ T cell penetration into the tumor core (≤100 CD8^+^ T cells/mm^2^) or stroma-restricted CD8^+^ T cells were associated with fibrotic or immunosuppressive tumor immune microenvironments, which predicted poor survival [22]. Thus, CD8^+^ T cell spatial localization is a predictor of clinical outcomes [22]. Supplemental Fig. 2 describes the classification of NOS2/COX2 strong single-cell intensity relative to the presence of CD8^+^ T cells in all tumors. Given the predictive power of CD8^+^ T cell spatial localization [22], we observed abundant stroma-restricted CD8^+^ T cells (Fig. 4C) with increased IFNγ expression (Fig. 4D) in regions proximal to elevated tumor NOS2 expression, indicating a potential association between CD8^+^ T cells, IFNγ, and NOS2 regulation (Fig. 4C, D). In contrast, Fig. 4E–H show areas with limited CD8^+^ T cells, as well as limited IFNγ and NOS2 expression. Importantly, COX2 is highly expressed in these regions (Fig. 4E–H). Pearson’s correlation coefficients were also determined and showed a significant correlation between tumor NOS2_hi_ expressing cells and CD8^+^ T cells/INFγ (Supplementary Fig. 3A). Interestingly, significant linearity was not observed between CD8^+^ T cells/IFNγ and tumor COX2 expression (Supplementary Fig. 3B). These results suggest that CD8^+^ T cells could provide a source of IFNγ leading to increased tumor NOS2 expression.

### High NOS2 cell niches, increased inflammation, and metastatic potential

**Fig. 5:**
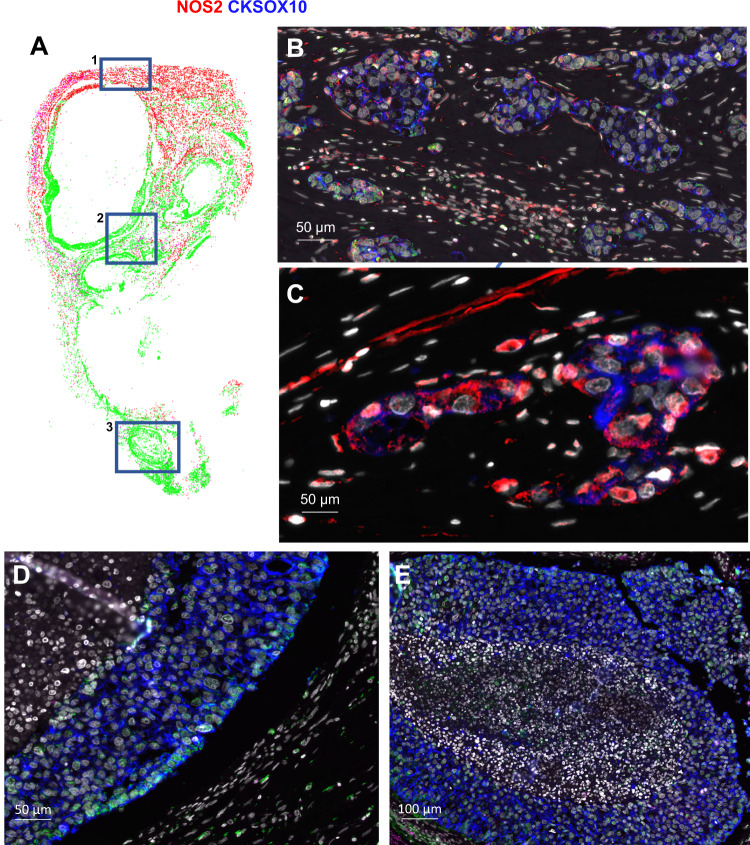
NOS2_hi_ regions are associated with increased satellitosis and metastatic potential. **A** Spatial distribution of the entire tumor with blue boxes labeled 1 (top), 2 (middle), and 3 (bottom) showing **B**, **C** magnification of the spatial localization of NOS2 (red) and the tumor marker CKSOX10 (blue) expressing cells in a NOS2_hi_ region (50 μm) highlighted within Box 1. Both elongated and clustered NOS2-expressing cells that have broken away from the larger lesion are shown, which is indicative of satellitosis and increased metastatic potential. These phenotypes are not observed in cold immune regions (**D**) or immune desert regions (**E**).

The stimulation of oncogenic pathways by NO is characterized by increased epithelial-to-mesenchymal transition (EMT), migration, and cancer cell motility culminating in cancer disease progression and metastasis [23, 24]. Patients in this cohort succumbed to metastatic disease even though lymph node-positive status was not observed at diagnosis. NOS2_hi_ regions were near stroma-restricted CD8^+^ T cells. NOS2_hi_ areas exhibited small tumor clusters that appeared to break away from the primary lesion (satellitosis), indicative of metastatic niches (Fig. 5A box 1, 5B, and 5C). In contrast, satellitosis was absent in regions with lower tumor NOS2 expression as well as fewer CD8^+^ T cells and IFNγ (Fig. 5D, E). These findings indicate that NOS2_hi_ clustering foci could promote increased metastatic potential, which is consistent with earlier reports [15, 25].

### Morphological changes that mimic NOS2^+^ niches

**Fig. 6:**
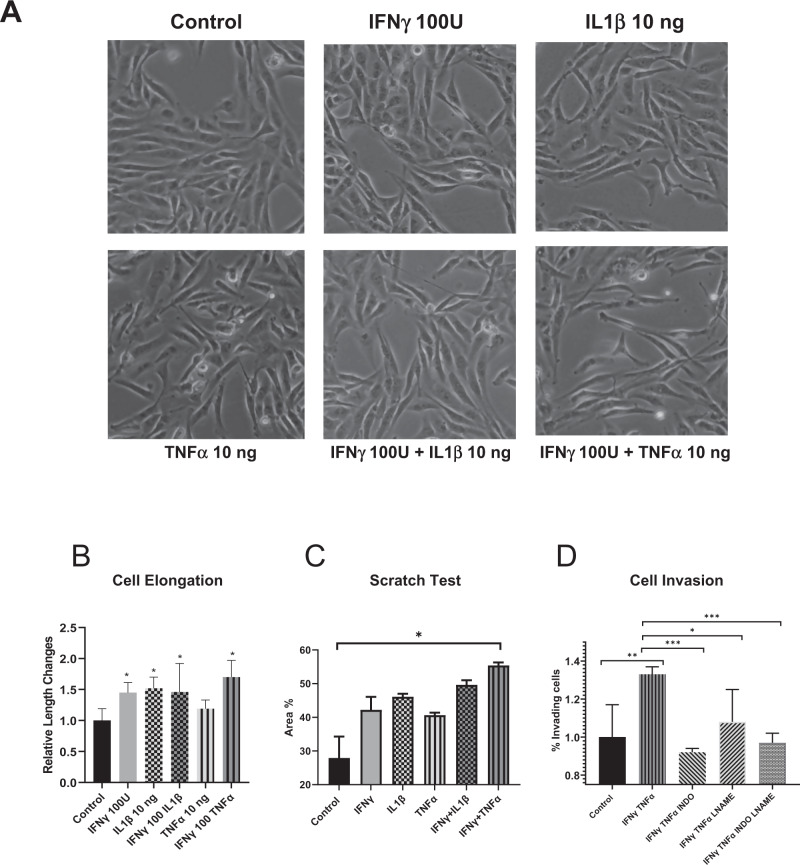
IFNγ and cytokines promote elongation and migration of MB231 breast cancer cells. **A** Microscope images (10X) of control and cytokine-treated MB231 cells for 48 h. **B** Cell elongation analysis after 48 h cytokine treatment. **C** A scratch test assay of MB231 cells after 12 h of cytokine treatment. **D** Cell invasion assay; MB231 cells were seeded in the upper well of a Boyden chamber with serum-free media ± cytokines and the pan-NOS/COX inhibitors LNAME and indomethacin for 48 h. The lower chamber was filled with complete media. Cells were counted against the standard curve. Results are presented as mean ± SD. *p < 0.05, **p < 0.001, ***p < 0.0001.

Cellular morphology is a key aspect of metastasis, where cells acquire an elongated phenotype during migration and invasion processes. In vitro migration models have shown NO roles during metastatic processes, where exposure to higher NO flux (100–300 nM) for 24–48 h increased in vitro migration and invasion of MB231 and MB468 breast cancer cells [7, 15]. These earlier observations suggest that increased tumor NOS2 expression and clustering would generate a flux of NO that enhances the metastatic potential of exposed cells within that niche [15]. Herein, we further explored the influence of tumor cell NOS2/COX2 expression on altered cellular morphology. As shown in Fig. 6A, B, MB231 cells exposed to individual, or combination cytokine treatment demonstrated morphological changes and cellular elongation characteristic of EMT in migrating and invading cells. In addition, scratch test assay showed increased wound closure after 12 h of IFNγ + TNFα combination treatment when compared to the untreated control cells (Fig. 6C). Similarly, Boyden chamber assays showed increased cell invasion in response to 48 h IFNγ + TNFα combination treatment, which was reduced by the pan-NOS/COX inhibitors (LNAME/Indomethacin) both as single treatment agents and in combination (Fig. 6D). These results suggest that the upregulation of NOS2/COX2 tumor expression within an inflammatory niche could generate phenotypes with increased metastatic potential (Fig. 6).

## Discussion

**Fig. 7:**
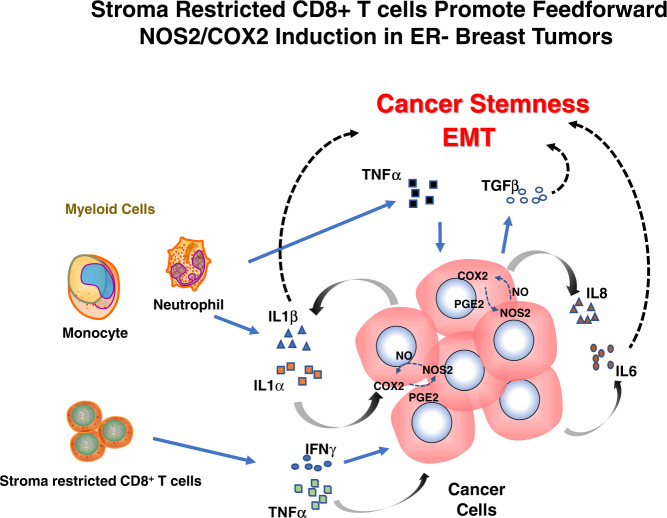
Interplay of cytokine production in the tumor microenvironment leading to NOS2_hi_/COX2_hi_ tumor expressing regions. The secretion of IFNγ by stroma-restricted CD8^+^ T cells and IL1β/TNFα secreted by myeloid cells within the tumor microenvironment leads to tumor NOS2/COX2 expression and the development of aggressive cellular niches with increased metastatic potential and promotes immunosuppression. Cellular neighborhoods expressing high tumor NOS2/COX2 then increase IL1α/β, creating a feedforward loop that maintains tumor NOS2/COX2 expression and elevated cytokines, including IL8 and IL-6 as well as the activation of latent TGFβ by NO. These factors conspire to promote immunosuppression, metastasis, and cancer stemness through NOS2-derived NO and COX2-derived PGE2.

One of the most effective prognostic indicators for ER- breast cancer is the association between NOS2 and COX2 [10, 26]. The data above demonstrates an unusual link between IFNγ and lymphoid cells in terms of clinical outcomes, as summarized in Fig. 7. IFNγ and CD8^+^ T cells are linked to and necessary for the production of high NOS2 and COX2 levels, despite the fact that they are predictive of positive clinical outcomes and a hallmark of a good prognosis in many malignancies [22, 27]. According to the scRNAseq results, IFNγ and IL1β/TNFα are necessary to induce the highest levels of NOS2 and COX2 expression, which suggests that lymphoid cells could be a contributing factor in the tumor. According to earlier research, IFNγ is critical for the stimulation of NOS2 in DLD1 (human colon cancer) cells [28]. Also, the scRNAseq of MB231 with IFNγ + IL1β reveals a strong connection between NOS2/COX2, and IL1β, TNFα, IL6, and IL8, bolstering the notion of a strengthened Th1 microenvironment acquired from the TCGA (Fig. 1). This implicates multifactor immune mechanisms leading to a feedforward loop that promotes the induction of high NOS2/COX2-expressing cellular niches and disease progression. Previous studies have demonstrated that IFNγ, IL6, PGE2, and IL1 all boosted NOS2 expression, suggesting that several reinforcing processes were involved in NOS2 upregulation [15]. In addition, IL1α is a mediator of ER stress that is frequently observed in the TME after chemotherapy. Both IL1α and ILβ increased in response to IFNγ and IL1β significantly enhances these complimentary pathways for the sustained elevation of NOS2/COX2 mechanisms. These factors could conspire to create a NOS2/COX2 inflammatory niche that promotes disease progression.

### Single-cell implications of NOS2 expression

Despite the protein’s sequence and biochemistry being comparable in the mouse and human, the NOS2 promoter is complicated and differs significantly between the two species [28–30]. Expression of NOS2 in murine macrophages maximally induced in vitro by IFNγ or LPS has been the gold standard in the NO field, where estimated NO flux could reach as high as 1–5 µM at the cellular level [13]. Murine tumor cells displayed significantly higher NOS2 activity in response to IFNγ or LPS, suggesting a key distinction between tumor cells and macrophages [13]. While data here clearly demonstrate that maximum human NOS2 expression is induced by IFNγ, IL1β, and TNFα, identical to that in murine tumor cells, human macrophages do not activate NOS2 with IFNγ or LPS. However, the levels of NO produced by mouse and human tumor cells still differ significantly on a fundamental level. Recently, it was demonstrated that the number of NOS2^+^ cells, rather than NOS2 expression, correlated with the amount of NO and nitrite produced in vitro [11]. As a result, the clusters of NOS2-expressing cells will affect NO levels, and NOS2 cell clustering can produce areas of greater NO flux [13, 31]. In vitro experiments reveal that high nitrite, and NO levels are present when 50–80% of the cells express NOS2 and fluxes are higher than 100 nM. However, NO production is an order of magnitude lower in 5% of human cancers. Our earlier research demonstrates that NO-driven carcinogenic pathways take place at an ideal concentration of 200–400 nM, which increases the expression of IL6 and IL8 [12]. Nonetheless, NO levels are higher where NOS2-expressing cells are concentrated at a much higher density, such as in localized foci inside the tumor. When considered collectively, these findings suggest that these regions of high-density NOS2-expressing cells have larger NO flux, which can trigger oncogenic mechanisms that take place in the range of 100–300 nM NO in the petri dish [32–34].

### The spatial configuration of the NOS2/COX2 niche

Areas enriched for CD8^+^ T cells and IFNγ are related to the juxtaposition of lymphoid and tumor cells within the TME, which results in high clustering of enhanced NOS2-expressing cells. An earlier study looking at CD8^+^ T cell placement demonstrated that spatial orientation is a crucial factor in the determination of TNBC clinical outcomes [22]. Positive results are described by tumor penetrating CD8^+^ T cells deep into the tumor core in a completely inflamed tumor. On the other hand, stroma-restricted CD8^+^ T cells and immune desert regions devoid of CD8^+^ T cells predict poor clinical outcomes [22]. Here we demonstrate that high NOS2 cellular niches can be formed at the tumor margin and proximal to stroma-restricted CD8^+^ T cells. These niches can now encounter IFNγ and other cytokines that induce tumor NOS2/COX2 expression. Contrarily, NOS2^+^ and COX2^+^ cells are scattered and observed at lower levels in areas with increased CD8^+^ T cell penetration into the tumor. The aforementioned information demonstrates unequivocally that CD8^+^ T cell and IFNγ are close to NOS2 and suggests that an inflammatory niche with stroma-restricted CD8^+^ T cells is necessary for NOS2 induction.

An immune desert devoid of CD8^+^ T cells is another significant aspect of the TME that has previously been identified [22]. Poor clinical outcome is suggested by low CD8^+^ T cell counts and exhausted CD8^+^ T cells in the tumor compartment [35]. One of the critical factors in the absence of CD8^+^ T cells associated with low IFNγ in immunological desert regions was also revealed in regions deficient in NOS2 with increased COX2 expression. This implies that the immunological desert is associated with COX2-positive and NOS2-negative regions. Elevated CD8^+^ T cells and other lymphoid cells that are restricted to tumor stroma or margins can result in situations that promote higher NOS2 and COX2.

### Ramification of NOS2/COX2 niche and metastatic potential

Our prior research demonstrated that NO is essential for promoting EMT and metastasis [15]. Increased inflammation at these NOS2 foci increases the likelihood of metastasis, and the discovery of the NOS2 positive niche at the tumor-stroma interface suggests that this may be the site of metastasis. Elongation and EMT induced by NO are known to mediate these effects [15]. Also, IL1 and PGE2 enhance EMT and cell motility in breast cancer [36, 37]. Herein, IFNγ and ILβ1/TNFα promote motility and elongation [7]. As a result, the NOS2/COX2 inflammatory niches increase the potential for cancer cell motility and metastatic spread. Therefore, limited metastatic potential can be achieved by inhibition of NOS2/COX2 feedforward loops [38]. Given that metastasis is the primary cause of cancer deaths, NOS2/COX2 spatial localization at these sites of inflammation could provide an early prognostic indicator of poor outcome even in the absence of lymph node-positive status [7].

### Summary

IFNγ plays a key role in the induction of proinflammatory antitumor immune responses [39]. However, recent studies have shown that IFNγ response is concentration dependent where low levels in the TME promote protumorigenic disease progression mediated in part through the downregulation of major histocompatibility complexes and upregulation of indoleamine 2,3-dioxygenase and programmed cell death ligand 1 [39]. In addition, IFNγ is necessary to stimulate tumor-specific NOS2/COX2 expression, which through a multifaceted process, also drives oncogenic pathways and shapes immunological profiles associated with poor prognosis [10, 11]. Given that IFNγ is secreted by cytolytic CD8^+^ T cells, spatial analysis suggests that the quantity and location of CD8^+^ T cells [22] present an opportunity for the formation of IFNγ regulatory processes within the TME, including the upregulation of tumor NOS2/COX2 expression and the development of niches that promote disease progression, metastasis, and poor clinical outcomes [7, 10, 11, 22].

## Materials and methods

### Cell culture

The MDA-MB231 (MB231) human breast cancer cell line was obtained from the American Type Culture Collection (ATCC, Manassas, VA) and grown in RPM1-1640 (Invitrogen) supplemented with 10% fetal bovine serum (FBS; Invitrogen, Waltham, MA) at 37 °C in a humidified atmosphere of 5% CO_2_ in the air. Cells were serum-starved overnight prior to experimentation. Depending on the downstream assays, the cells were incubated for 12, 24, or 48 h with the addition of ddH_2_O (control), IFNγ 100 U/mL (285-IF/CF, R&D Systems, Minneapolis, MN), IL1β 10 ng/mL (201-LB/CF, R&D Systems), TNFα 10 ng/mL (210-TA/CF, R&D Systems), IL17A 10 ng/mL (7955-IL-CF, R&D Systems), lipopolysaccharide (LPS, Sigma, St. Louis, MO) 10 mg/mL (L2630, Sigma), LNAME 500 mM (N5751, Sigma, St. Louis, MO), and/or Indomethacin 100 μM (I7378, Sigma, St. Louis, MO).

### In vitro scratch assay

One million cells were plated in a 60-mm dish and allowed to reach 100% confluency. A 200 μl pipette tip was used to etch a straight scratch line across the confluent monolayer. Floating and dead cells were eliminated by washing the dishes in 1X PBS, and then complete media was added. A 10x objective inverted microscope (EVOS, Life Technologies, Carlsbad, CA) was used to take images at 0, 4, 8, and 12 h. The open-source software ImageJ measured the pace at which scratch gaps are refilled (version 1.53 u) [40].

### Cell invasion assay

A cell invasion assay (cat# ab235697) in 96-well plate format from Abcam (Waltham, MA) was used. After cell synchronization, a complete medium was given to the lower chamber as an attractant, and 50,000 cells were seeded in the upper chamber with cytokines ± inhibitors for 48 h. Migrated fluorescent cells were counted at Ex/EM = 530/590 nm on a SpectraMax i3x plate reader (Molecular Devices, San Jose, CA) and compared to a standard curve made from the same cell line.

### Single-cell RNAseq

The single-cell library was generated using the 10x Genomics (San Francisco, CA) Single Cell 3’ Reagent Kit v3 and then sequenced in our sequencing facility (NCI at Frederick, MD) using an Illumina NovaSeq 6000. Sample cells in a suspension medium were examined for viability before library preparation. The cDNAs were sequenced after being barcoded, pooled, and amplified during the library preparation. On average, 10,000 cells per sample were sequenced. The Cell Ranger software provided raw reads as input (10x Genomics, Version 6.1.2). They were demultiplexed and converted into BCL files using the Cell Ranger. All readings were mapped to the human reference genome using the default 10x Genomics Pipeline (Version 3.1.0) after passing quality checks (GRCH38-30.0). Annotated transcript counts within each cell were used to construct UMI (Unique Molecular Identifier) count matrices.

### scRNAseq data analysis

The matrix h5 files for each sample were uploaded to the internal Partek (St. Louis, MO) Flow server for data processing and data mining. All counts were normalized using the default “counts per million, add 1, and log2 transformed” method. The GSA (gene-specific analysis) tool was then applied to discover genes that were differentially expressed between the various experimental samples. Absolute fold changes ≥2 and a *p* value <0.05 were used to select genes. We utilized the Loupe Browser (version 6.3.0, 10× Genomics) to visually inspect the aggregated and standalone datasets to analyze the clustering patterns of the scRNAseq data. To create correlation heat maps, scRNAseq datasets that had been directly processed in Seurat (version 4.0, Satija lab, https://satijalab.org/seurat/) from the Cell Ranger output were exported to RStudio (2022.07.2 Build 576, https://posit.co/) in parallel. Single-cell data is available upon request to the Corresponding Author.

### The Cancer Genome Atlas (TCGA) analysis

The breast cancer (BRCA) subset of TCGA (https://www.cancer.gov/about-nci/organization/ccg/research/structural-genomics/tcga) were accessed through the UCSC (University of California, Santa Cruz) Xena Browser (Date of access: 11/2/2022, https://xena.ucsc.edu/). In brief, all targeted Th1, Th2, Th17 cytokines, NOS2, and COX2 genes were surveyed in the Xena browser, and then exported to RStudio (2022.07.2 Build 576) for subsequent correlation analysis.

### Tissue collection and immunohistochemical analysis of patient tumor sections

Tumor specimens (*n* = 21) were obtained from breast cancer patients recruited at the University of Maryland (UMD) Medical Center, the Baltimore Veterans Affairs Medical Center, Union Memorial Hospital, Mercy Medical Center, and the Sinai Hospital in Baltimore between 1993 and 2003. Informed consent was obtained from all patients. The collection of tumor specimens, survey data, and clinical and pathological information (UMD protocol no. 0298229) was reviewed and approved by the UMD Institutional Review Board (IRB) for the participating institutions. The research was also reviewed and approved by the NIH Office of Human Subjects Research (OHSR no. 2248). Breast tumor NOS2 and COX2 expression was analyzed previously by IHC using 1:250 diluted NOS2 antibody and 1:50 diluted COX2 antibody (no. 610328 and 610204, respectively, BD Biosciences, San Diego, CA) and scored by a pathologist [7, 9]. For NOS2 staining, a combination score of intensity and distribution were used to categorize the immunohistochemical NOS2 stains where intensity received a score of 0–3 if the staining was negative, weak, moderate, or strong. The NOS2 distribution received scores of 0–4 for distributions <10%, 10–30%, >30–50%, >50–80%, and >80% positive cells [7]. For COX2 staining, scores of negative to weak [1, 2] or moderate to strong [3, 4] were categorized as low or high, respectively [9]. Herein, NOS2 and COX2 expressions were also analyzed by fluorescent staining performed on the Leica Biosystems (Wetzlar, Germany) Bond RX Autostainer XL ST5010 using the Bond Polymer Refine Kit (Leica Biosystems DS9800), with the omission of the Post Primary Block reagent, DAB and Hematoxylin. After antigen retrieval with EDTA (Bond Epitope Retrieval 2), sections were incubated for 30 min with COX2 (Cell Signaling Technology, Danvers, MA, no. 12282, 1:100), followed by the Polymer reagent and OPAL Fluorophore 520 (AKOYA, Marlborough, MA). The COX2 antibody complex was stripped by heating with Bond Epitope Retrieval 2. Sections were then incubated for 30 min with NOS2 antibody (Abcam no. ab15323, 1:50), followed by the Polymer reagent and OPAL Fluorophore 690. The NOS2 antibody complex was stripped by heating with Bond Epitope Retrieval 2 and then stained with CD8 (Abcam no. 101500, 1:100) or IFNγ (Abcam no. 231036, 1:200), followed by the Polymer reagent and OPAL Fluorophore 570. Sections were stained with DAPI and coverslipped with Prolong Gold Anti-Fade Reagent (Invitrogen). Images were captured using the Aperio ScanScope FL whole slide scanner (Leica). The original IHC previously reported [7, 9] and fluorescent NOS2/COX2 staining results were generally consistent.

Formalin-fixed paraffin-embedded (FFPE) tissue sectioned at 4 μm and mounted on SuperFrost Plus slides were stained with a FixVUE Immuno-8^TM^ Kit (formerly referred to as UltiMapper® kits (Ultivue Inc., Cambridge, MA), USA; CD8, NOS2, COX2, CKSOX10, and IFNγ cocktail) using the antibody conjugated DNA-barcoded multiplexed immunofluorescence (mIF) method [1]. These kits include the required buffers and reagents to run the assays: antibody diluent, pre-amplification mix, amplification enzyme and buffer, fluorescent probes and corresponding buffer, and nuclear counterstain reagent. Hematoxylin and Eosin (H&E) and mIF staining was performed using the Leica Biosystems BOND RX Autostainer. Before performing the mIF staining, FFPE tissue sections were baked vertically at 60–65 °C for 30 min to remove excess paraffin prior to loading on the BOND RX. The BOND RX was used to stain the slides with the recommended FixVUE (UltiMapper) protocol. During assay setup, the reagents from the kit were prepared and loaded onto the Autostainer in Leica Titration containers. Solutions for epitope retrieval (ER2, Leica Biosystems cat# AR9640), BOND Wash (Leica Biosystems cat# AR9590), along with all other BOND RX bulk reagents were purchased from Leica). During this assay, the sample was first incubated with a mixture of all four antibody conjugates, next the DNA barcodes of each target were simultaneously amplified to improve the sensitivity of the assay. Fluorescent probes conjugated with complementary DNA barcodes were then added to the sample to bind and label the targets; Next, a gentle signal removal step was used to remove the fluorescent probes of the markers. The stained slides were mounted in Prolong Gold Anti-Fade mountant (Thermo Fisher Scientific, Waltham, MA, cat# P36965 and coverslipped (Fisherbrand Cover Glass 22 × 40 mm, #1.5). Digital immunofluorescence images were scanned at 20× magnification. Images were co-registered and stacked with Ultivue UltiStacker software. The digital images were then analyzed using the HALO image analysis platform [41].

### Statistical analysis

Experiments were assayed in triplicate unless otherwise stated. Student *t*-test was employed to assess statistical significance using the GraphPad Prism software (version 9). Image analyses are reported as mean ± SEM and *T-*tests with Welch’s or Mann–Whitney correction were used when appropriate to determine significance. Linear analyses and Pearson’s correlations were also conducted to determine significant correlations between protein expressions using Prism software. Significance is reported as *p ≤ 0.05, **p ≤ 0.01, ***p ≤ 0.001, and ****p ≤ 0.0001. Single-cell correlation analyses were conducted in RStudio using the corrplot (0.92) in R (4.2.1).

## Supplementary information

Supplemental Figure Legends

Supplemental Figure 1

Supplemental Figure 2

Supplemental Figure 3

Authors Checklist

## Data Availability

Single-cell RNAseq data will be made available upon request.
