# Supplementary material for: Interferon-gamma is quintessential for NOS2 and COX2 expression in ER- breast tumors that lead to poor outcome
Source: Cell Death Dis. 2023 May 11;14(5):319. doi: 10.1038/s41419-023-05834-9 (PMC10175544; doi:10.1038/s41419-023-05834-9)
Supplement: Supplementary file 1 — Supplemental Figure Legends [file 41419_2023_5834_MOESM1_ESM.docx]

## Supplemental Figure Legends

## Supplemental Figure 1. NOS2 and COX2 spatial localization and signal intensity. A) The entire tumor was analyzed for strong, moderate, and weak NOS2 and COX2 fluorescence intensities at the single cell level in 21 NOS2_hi_/COX2_hi_ (n=10) and NOS2_lo_/ COX2_lo_ (n=11) tumor images and found to be consistent with the original IHC Pathologist scoring for area and intensity as reported (7, 9). B) Analysis of strong, moderate, and weak NOS2/COX2 signal intensities in Tumor vs Stroma regions are presented. NOS2 and COX2 fluorescent intensities were determined for each tumor using real-time tuning in HALO software, and then mean intensities and standard deviations (SD) were determined. Threshold intensities for weak, moderate, and strong expression levels were determined by adding 2, 4, or 6 SD, respectively to the mean intensity threshold setting. C) Pearson’s correlation coefficients and linear regression analyses were performed to determine statistically significant linear relationships for NOS2 vs COX2 tumor expression at strong, moderate, and weak signal intensities.

## Supplemental Figure 2. Classification of each tumor based on the single cell level of strong intensity NOS2/COX2 expression. The original IHC’s were designated NOS2/COX2 hi or low based upon Pathologist scoring of area and intensity of the entire tumor. Herein, the selection of population distribution between low and hi was used to determine NOS2/COX2 classification. These hi and low NOS2/COX2 population distributions were assigned as +/- to classify tumors based on the single cell NOS2/COX2 strongest thresholds and expressing cells as percentage of the total.  The designation of CD8^+^ T cells was determined as above or below the mean of 5%.

**Supplemental Figure 3.** Linear correlation analyses. Pearson’s correlation coefficients and linear regression analyses were performed to determine statistically significant linear relationships between Tumor NOS2^+^ vs CD8^+^ T cells/IFNγ (A) and Tumor COX2^+^ vs CD8^+^ T cells/IFNγ (B) in areas stratified for high intensity tumor NOS2/COX2 expression. Pearson’s r^2^ correlations, slopes and p values were determined and shown in each graph.
