# Supplementary material for: Interferon-gamma is quintessential for NOS2 and COX2 expression in ER- breast tumors that lead to poor outcome
Source: Cell Death Dis. 2023 May 11;14(5):319. doi: 10.1038/s41419-023-05834-9 (PMC10175544; doi:10.1038/s41419-023-05834-9)
Supplement: Supplementary file 2 — Supplemental Figure 1 [file 41419_2023_5834_MOESM2_ESM.pptx]

## Slide 1
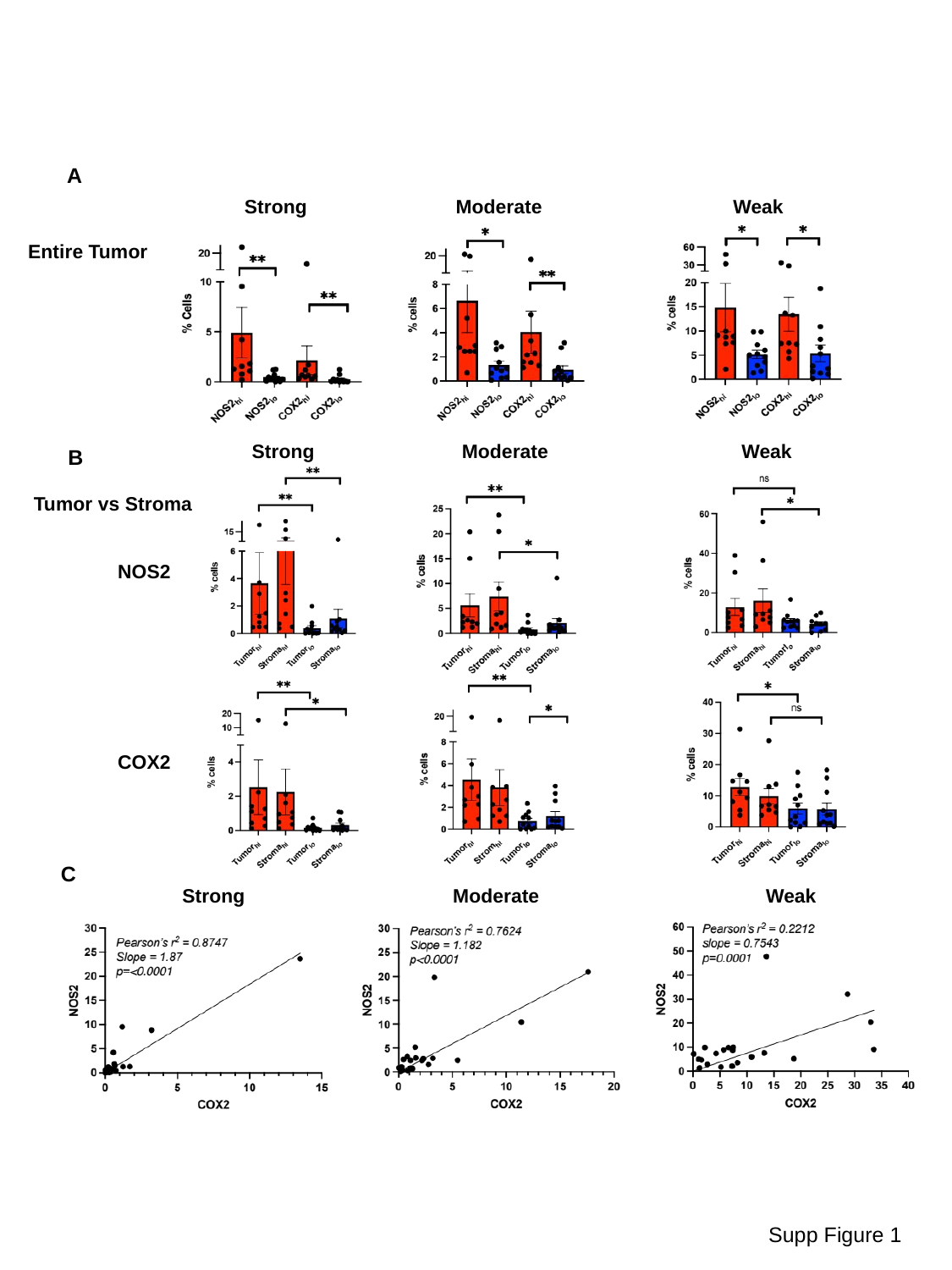

A
Strong
Moderate
Weak
Entire Tumor
Strong
Moderate
Weak
Tumor vs Stroma
NOS2
COX2
B
C
Strong
Moderate
Weak
Supp Figure 1
