# Supplementary material for: Interferon-gamma is quintessential for NOS2 and COX2 expression in ER- breast tumors that lead to poor outcome
Source: Cell Death Dis. 2023 May 11;14(5):319. doi: 10.1038/s41419-023-05834-9 (PMC10175544; doi:10.1038/s41419-023-05834-9)
Supplement: Supplementary file 3 — Supplemental Figure 2 [file 41419_2023_5834_MOESM3_ESM.pptx]

## Slide 1
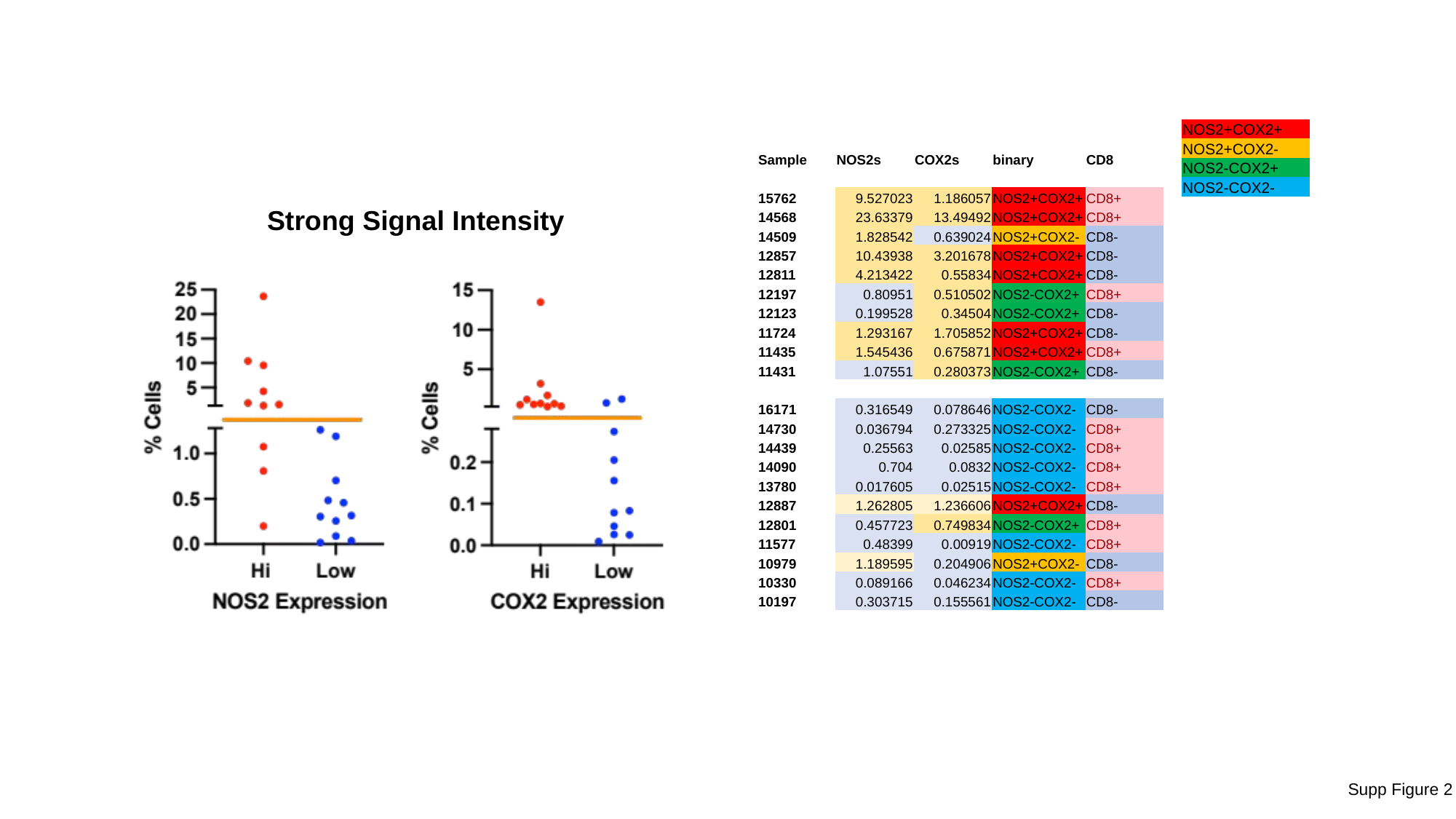

| |
| --- |
| NOS2+COX2+ |
| NOS2+COX2- |
| NOS2-COX2+ |
| NOS2-COX2- |
| Sample | NOS2s | COX2s | binary | CD8 |
| --- | --- | --- | --- | --- |
| | | | | |
| 15762 | 9.527023 | 1.186057 | NOS2+COX2+ | CD8+ |
| 14568 | 23.63379 | 13.49492 | NOS2+COX2+ | CD8+ |
| 14509 | 1.828542 | 0.639024 | NOS2+COX2- | CD8- |
| 12857 | 10.43938 | 3.201678 | NOS2+COX2+ | CD8- |
| 12811 | 4.213422 | 0.55834 | NOS2+COX2+ | CD8- |
| 12197 | 0.80951 | 0.510502 | NOS2-COX2+ | CD8+ |
| 12123 | 0.199528 | 0.34504 | NOS2-COX2+ | CD8- |
| 11724 | 1.293167 | 1.705852 | NOS2+COX2+ | CD8- |
| 11435 | 1.545436 | 0.675871 | NOS2+COX2+ | CD8+ |
| 11431 | 1.07551 | 0.280373 | NOS2-COX2+ | CD8- |
| | | | | |
| 16171 | 0.316549 | 0.078646 | NOS2-COX2- | CD8- |
| 14730 | 0.036794 | 0.273325 | NOS2-COX2- | CD8+ |
| 14439 | 0.25563 | 0.02585 | NOS2-COX2- | CD8+ |
| 14090 | 0.704 | 0.0832 | NOS2-COX2- | CD8+ |
| 13780 | 0.017605 | 0.02515 | NOS2-COX2- | CD8+ |
| 12887 | 1.262805 | 1.236606 | NOS2+COX2+ | CD8- |
| 12801 | 0.457723 | 0.749834 | NOS2-COX2+ | CD8+ |
| 11577 | 0.48399 | 0.00919 | NOS2-COX2- | CD8+ |
| 10979 | 1.189595 | 0.204906 | NOS2+COX2- | CD8- |
| 10330 | 0.089166 | 0.046234 | NOS2-COX2- | CD8+ |
| 10197 | 0.303715 | 0.155561 | NOS2-COX2- | CD8- |
| | | | | |
| | | | | |
| | | | | |
Strong Signal Intensity
Supp Figure 2
