# Supplementary figures and images for: Interferon-gamma is quintessential for NOS2 and COX2 expression in ER- breast tumors that lead to poor outcome
Source: Cell Death Dis. 2023 May 11;14(5):319. doi: 10.1038/s41419-023-05834-9 (PMC10175544; doi:10.1038/s41419-023-05834-9)

## Slide 1
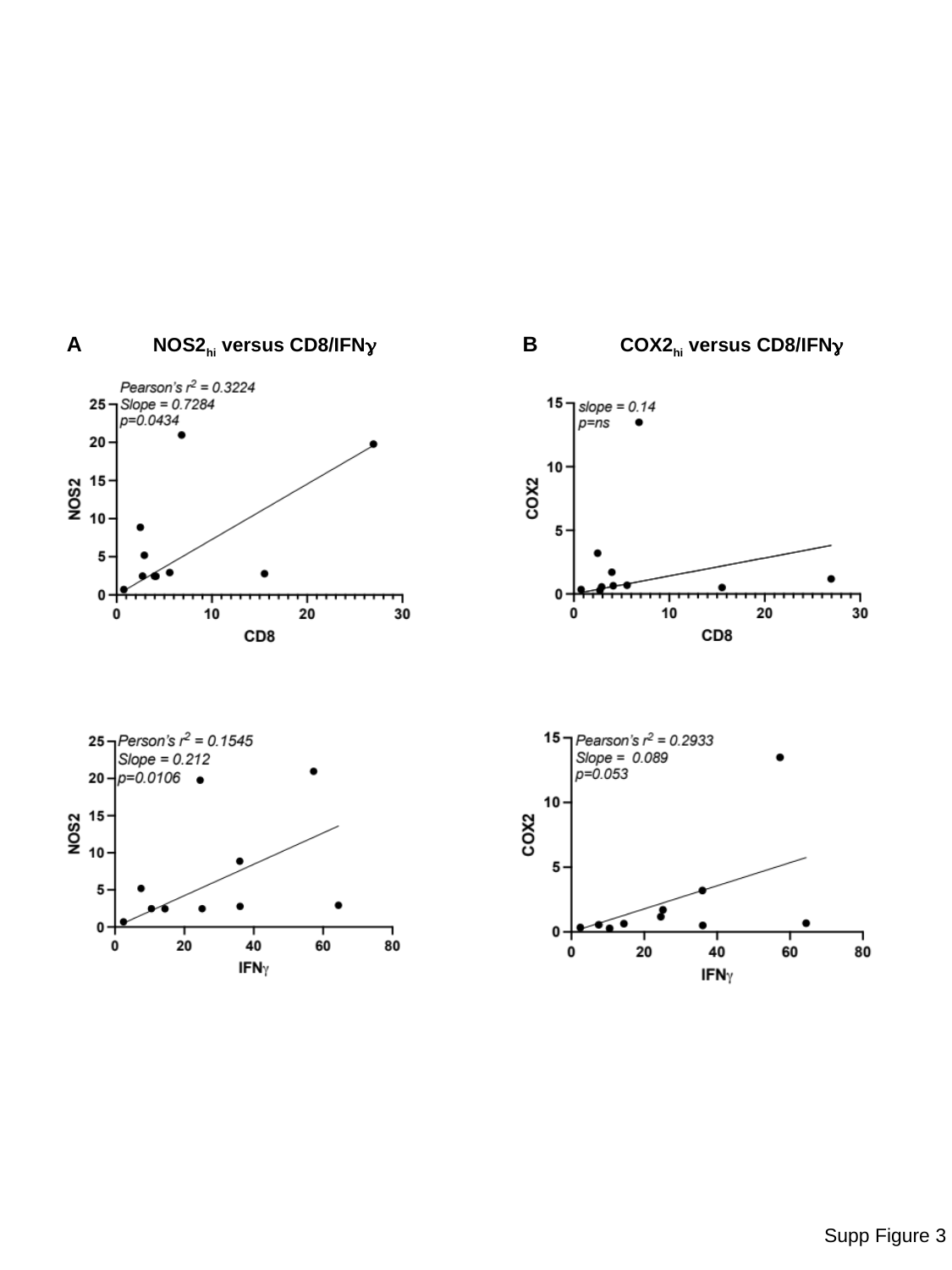

A NOS2hi versus CD8/IFNg
B COX2hi versus CD8/IFNg
All
Supp Figure 3

Supplement: Supplementary file 4 — Supplemental Figure 3 [file 41419_2023_5834_MOESM4_ESM.pptx]
